# Elementos de desinformação na percepção pública sobre a vacina contra a dengue no Brasil: uma análise de comentários em mídias sociais

**DOI:** 10.1590/0102-311XPT097124

**Published:** 2025-04-11

**Authors:** Iago Marafina de Oliveira, Roseni Pinheiro, Francisco Ortega, Emiliane Silva Santana

**Affiliations:** 1 Instituto de Medicina Social Hesio Cordeiro, Universidade do Estado do Rio de Janeiro, Rio de Janeiro, Brasil.; 2 Institució Catalana de Recerca i Estudis Avancats, Barcelona, España.; 3 Universitat Rovira i Virgilli, Tarragona, España.; 4 Escola Nacional de Saúde Pública da Bahia, Salvador, Brasil.

**Keywords:** Vacinas Contra Dengue, Percepção Pública da Ciência, Desinformação, Mídias Sociais, Dengue Vaccines, Public Perception of Science, Disinformation, Social Media, Vacunas Contra el Dengue, Percepción Pública da la Ciencia, Desinformación, Medios de Comunicación Sociales

## Abstract

Em 2024, a dengue no Brasil atingiu índices epidemiológicos alarmantes, sendo considerado como potencialmente o pior ano da história. Nesse contexto, a chegada de vacinas contra a doença representa um grande avanço para a saúde pública no país. Contudo, a desinformação e as notícias falsas sobre as vacinas representam uma ameaça ao êxito das campanhas de vacinação. Neste artigo, temos como objetivo compreender quais são os elementos de desinformação presentes na percepção pública sobre a chegada das vacinas contra a dengue no Brasil. Para isso, foi realizada a coleta de 1.550 comentários em postagens online de cinco grandes veículos de comunicação do país, resultando em cinco categorias temáticas criadas a partir de análise de conteúdo do tipo categorial: desinfodemia, vetores de desinformação, politização da vacina, hesitação vacinal/recusa vacinal e experiências pessoais. Conclui-se que quase 1/3 dos comentários coletados apresentam algum conteúdo desinformativo, o que reforça a necessidade de criação de diferentes estratégias de enfrentamento e combate à desinformação. O cenário de hesitação vacinal e politização de assuntos relacionados à saúde pública deixado pela COVID-19 é de grande relevância para discussões na esfera pública e um desafio a ser enfrentado nos próximos anos.

## Abstract (en)

In 2024, dengue in Brazil reached alarming epidemiological rates, being considered potentially the worst year in history. In this context, the arrival of vaccines against the disease represents a great advance for Brazil’s public health. However, misinformation and fake news about vaccines pose a threat to the success of immunization campaigns. In this article, we aim to understand the elements of misinformation present in the public perception about the arrival of dengue vaccines in Brazil. To this end, 1,550 comments were collected on online posts from five major media outlets in the country, resulting in five thematic categories created from a categorical content analysis: disinfodemic, misinformation vectors, vaccine politicization, vaccine hesitancy/vaccine refusal, and personal experiences. It is concluded that almost 1/3 of the comments collected have some misleading content, which reinforces the need to create different strategies to confront and combat misinformation. The scenario of vaccine hesitancy and politicization of public health-related issues that exist as a consequence of COVID-19 is of great relevance for discussions in the public sphere and a challenge to be faced in the coming years.

## Resumen

En 2024, el dengue alcanzó altas tasas epidemiológicas en Brasil, considerado como potencialmente el peor año de la historia. En este contexto, las vacunas contra la enfermedad representan un gran avance para la salud pública en el país. Sin embargo, la desinformación y las noticias falsas sobre las vacunas son una amenaza para el éxito de las campañas de vacunación. Este artículo tuvo por objetivo identificar cuáles son los elementos de desinformación presentes en la percepción pública sobre la llegada de las vacunas contra el dengue a Brasil. Para ello, se recopilaron 1.550 comentarios en publicaciones en línea de cinco de los principales medios de comunicación del país, lo que dio como resultado cinco categorías temáticas a partir del análisis de contenido categórico: desinfodemia, vectores de desinformación, politización de vacunas, vacilación/rechazo vacunal y experiencias personales. Se concluye que casi 1/3 de los comentarios recopilados tenían algún contenido de desinformación, lo que refuerza la necesidad de crear diferentes estrategias para enfrentar y luchar contra la desinformación. El escenario de vacilación vacunal y de politización de los problemas de salud pública provenientes de la COVID-19 fue de gran relevancia para las discusiones en la esfera pública y un desafío para enfrentar en los próximos años.

## Introdução

A dengue representa uma ameaça significativa à saúde pública, especialmente em regiões tropicais e subtropicais, onde condições ambientais propiciam a proliferação do mosquito *Aedes aegypti*, vetor transmissor do vírus ^1^. Surtos de dengue no Brasil são recorrentes há décadas, mas 2024 atingiu índices epidemiológicos alarmantes, sendo considerado pela Organização Pan-Americana da Saúde (OPAS) como potencialmente o pior da história, com um aumento de 291% no número de casos quando comparados aos números do primeiro trimestre de 2023 ^2^. A complexidade do controle da dengue é agravada pela ausência de tratamento específico e pela necessidade de intervenções focadas na prevenção, como a eliminação de criadouros de mosquitos e campanhas de conscientização para a população, o que ressalta a importância de estratégias de comunicação em saúde eficientes. 

A chegada de vacinas contra a dengue no Sistema Único de Saúde (SUS) representa um grande avanço. A Dengvaxia, primeira vacina autorizada pela Agência Nacional de Vigilância Sanitária (Anvisa) em 2015, foi produzida pelo laboratório francês Sanofi, mas passou a ser contraindicada por apresentar risco nas primeiras infecções, caso os vacinados contraíssem o vírus, como foi constatado nas Filipinas, onde vacinados contraíram doenças mais graves, gerando mais desconfiança nas instituições públicas de saúde e colaborando para a baixa cobertura vacinal em relação a outras vacinas ^3^. Recentemente, chegou ao SUS um segundo imunizante, o Qdenga, produzido pelo laboratório japonês Takeda Pharmaceutical Company, que foi recebido com entusiasmo por especialistas ^4^. Apesar de ser uma novidade promissora, a chegada das vacinas contra a dengue expõe desafios importantes em um contexto de desinformação, polarização política e presença de movimentos antivacina no espaço público resultantes da governança da pandemia de COVID-19 durante o governo de Jair Messias Bolsonaro ^5^. Nesse sentido, este artigo procura responder a seguinte pergunta: quais são os principais elementos de desinformação presentes na percepção pública sobre a chegada da vacina contra a dengue no Brasil?

A literatura científica descreve que a aceitação e adesão da população às campanhas de vacinação são cruciais para o êxito da cobertura vacinal ^6^. Em tempos de uma veloz disseminação de informação, destaca-se a importância de compreender a percepção pública sobre ciência e tecnologia para o desenvolvimento, a execução e a implementação de novas campanhas e políticas de saúde ^7^. Estudos anteriores demonstraram que as atitudes, crenças e preocupações da população podem influenciar significativamente a aceitação de intervenções e cobertura vacinal, afetando diretamente a efetividade das campanhas ^7^. Além disso, a comunicação em saúde desempenha um papel crucial na construção da confiança e na disseminação de informações precisas, especialmente em um contexto de infodemia ^8^ ^,^ ^9^ ^,^ ^10^.

Apesar da ampla discussão existente sobre a importância da comunicação em saúde, no Brasil e no mundo, especialmente no contexto de vacinação, ainda são poucos os estudos que exploram a percepção pública em relação à vacina contra a dengue no país em virtude da recente chegada do imunizante ao território brasileiro ^11^. Nesse contexto, a investigação dos comentários postados em artigos jornalísticos de grandes veículos de comunicação do Brasil emerge como uma oportunidade valiosa para explorar as opiniões, preocupações e expectativas da população em relação a essa nova intervenção de saúde pública. Uma análise recente traça a transformação da pesquisa em comunicação *online* no país em três fases diferenciadas ^12^. Inicialmente, as redes sociais foram vistas como agentes de transformações sociais. Em seguida, o desenvolvimento de estudos na área consolidou uma diversidade de abordagens e métodos. Por fim, a partir da década de 2010, as pesquisas no campo começam a reconhecer os riscos das redes sociais como propagadoras de desinformação, destacados em eventos como as jornadas de 2013 e a eleição presidencial de 2018. 

Diante desse contexto, a análise dos comentários em artigos jornalísticos mostra-se relevante para compreender aspectos fundamentais da percepção pública. Este estudo busca analisar os principais elementos de desinformação presentes na percepção pública neste momento de chegada da vacina contra a dengue no Brasil por meio dos comentários publicados em postagens jornalísticas na rede social Facebook. Ao todo, foram analisados 1.550 comentários em perfis de cinco grandes portais jornalísticos nos dois primeiros meses de 2024, o que gerou a criação de cinco categorias temáticas geradas por meio de análise de conteúdo do tipo categorial ^8^: desinfodemia, vetores de desinformação, polarização política da vacina, hesitação vacinal/recusa vacinal e experiências pessoais.

## Método

Este estudo é de natureza qualiquantitativa, criando um protocolo de pesquisa com abordagem mista para coleta de dados e interpretação de resultados. Para isso, foi iniciada uma revisão exploratória de literatura no campo dos estudos sobre comunicação e saúde. Grande parte dos estudos encontrados sobre desinformação e vacinas integram pesquisas e revisões bibliográficas sobre o caso da COVID-19, mas fornecem elementos importantes no que tange aos eixos como desinfodemia, hesitação vacinal e polarização política de vacinas ^13^ ^,^ ^14^ ^,^ ^15^ ^,^ ^16^. Já os estudos que traçam análises sobre comunicação e vacinas da dengue ainda são incipientes, visto a descontinuidade do primeiro imunizante em 2015 e a atualidade do tema no que se refere ao imunizante recém-chegado ao país. De todo modo, foi possível reunir estudos que investigam campanhas de prevenção a arboviroses, como dengue, Zika e chikungunya ^17^ ^,^ ^18^ ^,^ ^19^ ^,^ ^20^ ^,^ ^21^ ^,^ ^22^.

Essa primeira etapa auxiliou a conhecer o estado da arte sobre o tema a ser discutido e criar o livro de códigos para a análise de conteúdo ^23^. Os cinco principais códigos (C) são oriundos dos temas mais presentes nos artigos encontrados na revisão citada anteriormente, sendo adaptados como: (C1) desinfodemia; (C2) vetores de desinformação; (C3) polarização política da vacina; (C4) hesitação vacinal e recusa vacinal; e (C5) experiências pessoais. Em outras palavras, os códigos foram criados com auxílio da revisão da literatura para posterior leitura e codificação de cada comentário. A coleta de dados no Facebook foi realizada por uma pesquisadora, enquanto a codificação dos comentários, segundo as categorias códigos criados, foi realizada por dois pesquisadores. O Quadro 1 representa a relação entre a revisão de literatura e a criação do livro de códigos que permitiu interpretar os dados coletados.

**Quadro 1:** Relação entre códigos de análise e literatura.

CÓDIGO	ESTUDO (ANO)
C1 Desinfodemia	Pereira Neto et al. ^13^ (2022); Floss et al. ^14^ (2023); Henriques ^15^ (2018); Garcia & Duarte ^16^ (2020)
C2 Vetores de desinformação	Pereira Neto et al. ^13^ (2022); Floss et al. ^14^ (2023); Henriques ^15^ (2018); Garcia & Duarte ^16^ (2020)
C3 Polarização política da vacina	Pereira Neto et al. ^13^ (2022); Floss et al. ^14^ (2023); Henriques ^15^ (2018); Garcia & Duarte ^16^ (2020)
C4 Hesitação vacinal e recusa vacinal	Sato ^37^ (2018); Posetti & Bontcheva ^29^ (2020); Abe et al. ^30^ (2012); Vasconcellos-Silva ^31^ (2022); Risi Júnior ^43^ (2003); Cunha ^44^ (2022)
C5 Experiências pessoais	Andrade et al. ^17^ (2020); Campos & Corrêa ^18^ (2019); Mafra & Antunes ^19^ (2015); Rangel-S ^20^ (2008); Rumpf ^21^ (2019); Valle et al. ^22^ (2016)

Fonte: elaborado pelos autores.

Após a criação do livro de códigos, optamos por realizar a pesquisa analisando os comentários de usuários em postagens de portais de notícias no Facebook. Essa rede social foi selecionada devido à sua capacidade avançada de pesquisa. Ao contrário do Instagram, por exemplo, que não oferece uma ferramenta de busca de descritores dentro dos perfis, o Facebook permite a filtragem de postagens por data e palavras-chave. Isso facilitou significativamente a localização de postagens relevantes para nossa análise, colaborando para um processo mais eficiente e preciso de coleta de dados.

Apoiados na metodologia da análise de conteúdo de Bardin, especificamente a do tipo categorial, cumprimos as seguintes etapas ^23^ ^,^ ^24^ ^,^ ^25^ ^,^ ^26^: (a) seleção das páginas/portais, (b) criação das unidades amostrais, ou seja, as notícias publicadas em um recorte temporal; (c) coleta dos dados; (d) aplicação do livro de códigos aos dados coletados; (e) análise dos comentários codificados; (f) discussão crítica dos resultados. O critério para a seleção dos jornais e portais de notícias foi o maior número de seguidores em suas respectivas páginas no Facebook, a saber: G1 (11 milhões), UOL Notícias (6,4 milhões), O Globo (5,6 milhões), Folha de S.Paulo (5,3 milhões) e Estadão (3,6 milhões). O número de seguidores foi adotado como critério de inclusão em virtude da maior abrangência das postagens, levando a uma base de dados maior. 

Além disso, escolhemos coletar os comentários somente dentro de suas respectivas postagens no Facebook e optamos por não incluir possíveis comentários dentro do site de notícias para as quais as postagens direcionaram. Isso se deu porque em muitos deles há a necessidade de cadastro do usuário, muitas vezes associada a uma taxa de assinatura que pode atuar como uma barreira para participação, as conhecidas *paywalls* que dificultam a leitura na íntegra das matérias. Foi utilizado como recorte temporal os últimos cinco anos, de 2019 a 2024, na tentativa de compreender a repercussão sobre o desenvolvimento da vacina. Contudo, não encontramos postagens anteriores a 2022. Os descritores utilizados foram “vacina” AND “dengue”. Ao todo, foram encontradas 49 postagens até fevereiro de 2024, data da escrita deste artigo, conforme a Tabela 1.

**Tabela 1:** Resultados das buscas.

Jornal/Portal	Notícias	Comentários	Média de engajamento
G1	7	468	66,85
UOL Notícias	6	302	50,33
Folha de S.Paulo	7	78	11,14
O Globo	20	561	28,05
Estadão	9	141	15,67
Total	49	1.550	34,00

Fonte: elaborado pelos autores.

O número total de comentários compõe a amostragem da pesquisa, do tipo não probabilística por relevância, justamente pelas unidades textuais (os comentários) não se constituírem por unidades indivisíveis ^24^. Isso quer dizer que os comentários selecionados foram analisados à luz do objetivo e da pergunta de pesquisa. Por fim, é importante destacar que os comentários foram transcritos para as planilhas, de forma literal, mas sem o nome dos autores a fim de preservar a sua identidade e respeitar aspectos éticos. Como se trata de comentários públicos em páginas abertas e não houve interação entre os pesquisadores e os sujeitos, não foi necessária apreciação do estudo em comitê de ética em pesquisa.

## Resultados e discussão

Após a aplicação dos códigos, o total de comentários coletados (1.550) transformou-se em uma amostragem de 461 comentários identificados com algum dos elementos de desinformação, conforme os critérios de objetivo e pergunta de pesquisa. Esse é um dado importante, já que esses comentários correspondem a 29,74% do total de comentários publicados nas postagens.

A pesquisa codificou 461 comentários, a saber: desinfodemia (C1 = 63), vetores de desinformação (C2 = 30), polarização política da vacina (C3 = 232), hesitação vacinal e recusa vacinal (C4 = 94) e experiências pessoais (C5 = 42). A Tabela 2 demonstra a amostragem codificada.

**Tabela 2:** Amostragem codificada.

Jornal/Portal	Comentários				
	C1	C2	C3	C4	C5
G1	43	5	66	32	16
UOL Notícias	4	4	25	23	15
Folha de S.Paulo	3	2	19	7	1
O Globo	13	19	61	28	9
Estadão	0	0	61	4	1
Total	63	30	232	94	42

Fonte: elaborado pelos autores.

Nota: C1: desinfodemia; C2: vetores de desinformação; C3: polarização política da vacina; C4: hesitação vacinal e recusa vacinal; e C5: experiências pessoais.

Cada um dos códigos gerou categorias de análise que foram discutidas criticamente em revisão de literatura. No Quadro 2, selecionamos a título de exemplo cinco comentários de cada categoria que elucidam a amostragem do conteúdo analisado e estão reproduzidos na íntegra [sic].

**Quadro 2:** Exemplos de comentários [sic].

CÓDIGO	COMENTÁRIOS
C1	“*Oxe eu fiquei sabendo que só podia tomar vacina quem nunca teve dengue eu não entendi agora essa*” (Trecho 1 - C1)
	“*Nem Brasil, Índia e China que tem doenças tropicais desenvolveram essa vacina e vão confiar no Japão, um país que não tem essa doença e muito menos tradição vacinal. A vac do Covid tem efeito até hoje: ataque cardíaco de jovens e trombose enfim... Japão pode até desenvolver vacina pra outros países mas não confio*” (Trecho 2 - C1)
	“*Nunca vi ou ouvi dizer que crianças tem dengue. Se tiver são raro pq a vacina pra essa faixa etária. Querem acabar com sistema imunológico só pode viu*” (Trecho 3 - C1)
	“...*mentira, pare de se enganar, não tem vacina, e o governo não vai comprar, e muita gente vai morrer de dengue, tudo por causa do genocida do Lula*” (C1)
	“*Enquanto em 2020 até 2022 o mosquito modificado geneticamente da Dengue tirou férias para a Covid assumir, só idiota alienado/adestrado cognitivo compra isto de novo*” (Trecho 5 - C1)
C2	“*Agora a turminha do ‘L’ está vendo claramente e sentindo na pele o que é o DESASTROSO DESGOVERNO do COMUNISTA MENTIROSO e GATUNO descondenado LULA. BATE FORTE com a mão no peito e FAÇA um ‘L’ com muito orgulho para FALTA de VACINA contra DENGUE no SUS, graças ao REI dos IMPOSTOS e FAMINTO DEVORADOR de DINHEIRO PÚBLICO LULA, PREJUDINCANDO cada dia mais os BRASILEIROS. Esquerda sendo esquerda! Hilário!* [link para o portal O Antagonista]” (Trecho 1 - C2)
	“*A IMPRENSA ESTÁ SENDO PAGA PARA TE MANIPULA, DUVIDA? OLHA QUANTO GOVERNO ESTÁ GASTANDO COM PUBLICIDADE! IMPRENSA, TV, RECEBENDO MUITO ATRAVEZ DE PROPAGANDA ESTATAIS. LEIA É MUITO IMPORTANTE PARA VOCÊ E SUA FAMÍLIA! VOCÊ ACORDE ANTES QUE SEJA TARDE, GOVERNO DO PT LULA ESTÁ SÓ AUMENTANDO O IMPOSTO, TAXAS, DESEMPREGO ESTÁ SUBINDO E A imprensa está sendo paga com verba de publicidade para fazer povo de otario, idiotas, com narrativas, informações distorcida e manipuladas* (...) *Caso não acredite em mim, O PAÍS ESTÁ FALINDO, olhem como está o nordeste. É SERIO MUITAS EMPRESAS JÁ ESTÃO SE INDIVIDANDO E FALINDO, MUITOS DONOS DE IMÓVEL NÃO ESTÃO CONSEGUINDO ALUGA OU VENDE SEUS IMÓVEIS, PREÇO DOS ALIMENTOS ESTÃO SUBINDO, ACORDEM LOGO* [três links para vídeos na plataforma YouTube, sendo dois deles a um canal ligado ao anarcocapitalismo]” (Trecho 2 - C2)
	“[link de publicação no X, antigo Twitter] *Parabéns sistema globolixo, hoje gananciosos e sabotadores, pela reportagem na época em que surfavam nas graças da gigantesca aceitação e popularidade da LAVA-JATO junto à imensa parcela da sociedade brasileira de caráter e conduta moral*” (Trecho 3 - C2)
	“*GOVERNO DO DESCASO, GOVERNO DA ROUBALHEIRA* [link para publicação com informação falsa no próprio Facebook]” (Trecho 4 - C2)
	“*A ‘justiça canhota’ está fazendo CENSURA de vídeo que mostra a OSTENTAÇÃO MILIONÁRIA do filho do juiz Benedito “TAPINHA DO LULA NA CARA” Gonçalves em Amesterdã, Holanda, porque, segundo a juíza que proferiu a CENSURA, o vídeo ridiculariza o pai que juiz da STJ, sem que nenhuma irregularidade jurídica tenha sido cometida. Agora é oficial, a CENSURA voltou, quando algum juiz não gostar de algum vídeo ou conteúdo midiático, ele vai fazer CENSURA e mandar retirar o conteúdo de circulação, mesmo sem respaldo jurídico. Está até parecendo o XERIFE XANDÃO, acho que ela aprendeu com o SINISTRO do STF. Esquerda sendo esquerda! Hilário!* [link para o portal O Antagonista]” (Trecho 5 - C2)
C3	“*AAAHH Se fosse o governo Bolsonaro! A narrativa seria: falta empenho desse governo em conseguir mais doses da vacina. Mas como se trata do governo que essa mesma imprensa, ajudou a eleger; silêncio total*” (Trecho 1 - C3)
	“*Uma população de mais de 200 milhões de habitantes, essas doses estão perto de atender a população, já pensou se o presidente fosse o Bolsonaro como seria noticiado pelo g1?*” (Trecho 2 - C3)
	“*Foi feita alguma ação/campanha contra a dengue pelo governo federal no ano passado? Parece que ele tirou dinheiro dos Estados, que por sua vez retiraram ações básicas como o fumacê! Já pode chamar o governo de genocida?*” (Trecho 3 - C3)
	“*E o Desgoverno Lula não quer comprar a vacina da dengue. E os esquerdobostas não dizem um ‘a’ desse genocídio*” (Trecho 4 - C3)
	“*Lula, o pai da dengue. O LADRÃO bateu outro recorde negativo em 2023 Maior número de óbitos por causa da DENGUE. #fazueliquepassa*” (Trecho 5 - C3)
C4	“*VACINAS. NÃO TOMO VACINAS EM HIPÓTESE ALGUMA. COM VACINA, AGORA É QUE O POVO VAI RELAXAR MESMO NAS IMUNDÍCIES*” (Trecho 1 - C4)
	“*Eu não vou tomar. Ninguém manda em mim agora eu vou posso escolher não morre do mesmo jeito pra que vou tomar*” (Trecho 2 - C4)
	“*Que se ferre as ‘vacinas’. Depois dessas ‘vacinas’ do covid eu passei a desconfiar de toda substância disponibilizada pelo Estado, por governantes. Me sinto salvo por não ter tomado essas vacinas do covid. E estou fora de qualquer outra que venha por aí*” (Trecho 3 - C4)
	“*Espero que o Ministério da Saúde, reveja decisão de não comprar essa vacina*” (Trecho 4 - C4)
	“...*vender vacina e obrigar as pessoas a tomarem 500 doses é uma combinação perfeita.* *eu não sou contra as vacinas, sou contra esse mecanismo cleptocrático*” (Trecho 5 - C4)
C5	“...*na minha família tem uma professora que ficou Paralítica pela picada desse Mosquito*” (Trecho 1 - C5)
	“*Aqui em Anápolis Goiás ninguém nem usa mais o transporte público, os mosquitos estão carregando geral*” (Trecho 2 - C5)
	“...*o meu irmão teve e as plaquetas ñ subiram nunca mais.* *Não pode nem tomar corticoides*” (Trecho 3 - C5)
	“*A morte que houve na minha cidade não está contabilizada aí*” (Trecho 4 - C5)
	“*As crianças que morreu ano passado em MG e forisso sintoma de dengue e mídias e mentiram pra não alarma foco de dengue nas escolas… governo de MG e genocídio*” (Trecho 5 - C5)

Fonte: elaborado pelos autores.

Nota: C1: desinfodemia; C2: vetores de desinformação; C3: polarização política da vacina; C4: hesitação vacinal e recusa vacinal; e C5: experiências pessoais.

### Desinfodemia (C1)

A categoria “desinfodemia” (C1) teve o terceiro maior número de comentários obtidos pela pesquisa e inclui 63 comentários que continham algum tipo de desinformação sobre as novas vacinas contra a dengue. É mister, nesse primeiro momento, ressaltar que infodemia é um termo que faz a junção das palavras “informação” e “epidemia”, retratando um contexto em que as informações são propagadas de maneira cada vez mais rápida, como um vírus ^27^. O termo infodemia tem origem em 2003 e foi criado pelo jornalista David J. Rothkopf durante a epidemia de SARS ^28^. O fenômeno não é novo, mas devido a transformações tecnológicas nos últimos anos, ele foi potencializado. É importante ressaltar que a infodemia se refere apenas à rápida e crescente circulação de informação, seja ela factível ou não. Nesse sentido, o termo desinfodemia aplica-se especificamente à infodemia de desinformação, ou seja, a grande e rápida circulação de *fake news* ^29^.

Ao mesmo tempo, o Quadro 2 permite compreender diferentes níveis de desinformação, o que coaduna com as três distinções conceituais de desinformação retratadas na literatura ^13^. A desinformação (*disinformation*) refere-se à criação e propagação deliberada de informações falsas capazes de produzir prejuízos a indivíduos, grupos, organizações e até mesmo países. Esse é o tipo que mais se aproxima da noção de *fake news* - e tem origem no período da Guerra Fria, sendo descrita no *Chambers Twentieth Century Dictionary* em 1972 como um vazamento proposital de informações falsas ^14^. Já *misinformation* é o tipo de desinformação que engloba as informações falsas que não são repassadas necessariamente com o intuito de produzir danos, o que acontece quando sujeitos compartilham *fake new*s achando que estão fazendo algo bom. Por fim, a informação maliciosa (*malinformation*) trata das informações baseadas na realidade, mas que são descontextualizadas com a intenção de produzir danos. Nos comentários selecionados para ilustrar esta seção, é possível perceber tais distinções levando em consideração a falsidade do conteúdo propagado e o grau de danos capazes de serem gerados ^14^ ^,^ ^15^ ^,^ ^16^.

Os comentários codificados na análise expressam as três distinções de desinformação. No Quadro 2, é possível perceber confusão sobre os critérios de elegibilidade para a vacinação contra a dengue, sugerindo que apenas aqueles que nunca contraíram a doença poderiam ser vacinados. Essa percepção equivocada pode levar a uma subutilização da vacina, baixando a cobertura vacinal e aumentando o risco de surtos de dengue, já que a vacinação é uma ferramenta importante para prevenir a doença ^4^. Outros comentários desinformam sobre a vacina, citando a desconfiança no país produtor, o Japão. Essas alegações infundadas minam a confiança nas vacinas em geral e podem levar indivíduos a recusarem a imunização, comprometendo assim os esforços de saúde pública para o controle de doenças. Além disso, alguns comentários refletem crenças de que crianças não são afetadas pela dengue e, portanto, não precisam ser vacinadas, o que negligencia o fato de que crianças podem contrair dengue e desenvolver complicações graves, levando inclusive à óbito, destacando a importância de informar o público sobre a importância da vacinação em todas as faixas etárias contempladas, conforme esquema vacinal ^3^ ^,^ ^4^ ^,^ ^30^.

A propagação de teorias da conspiração também foi evidente em alguns comentários, com alegações infundadas de que o governo não comprará a vacina da dengue e de que a vacinação de crianças está associada a efeitos colaterais graves, como o acidente vascular cerebral (AVC). Essas teorias da conspiração alimentam o medo e a desconfiança em relação às autoridades de saúde e às vacinas, prejudicando o combate a doenças que sobrecarregam a própria capacidade de atenção no SUS, um fenômeno em crescimento desde a pandemia de COVID-19, momento no qual o fanatismo e teorias da conspiração prejudicaram tanto a adesão às estratégias de prevenção quanto às campanhas de vacinação ^5^ ^,^ ^31^. 

Essa categoria auxilia a compreender, na prática, como a desinformação não é apenas prejudicial à saúde individual, mas também à saúde pública, uma vez que um plano de imunização é entendido como pacto coletivo. Quando informações incorretas são disseminadas em grande escala, elas podem minar a confiança nas instituições de saúde, reduzir a adesão às campanhas e a capacidade de gestão de controle de doenças, aumentando o risco de surtos e epidemias ^32^. É crucial o investimento em educação em saúde e alfabetização digital ^33^. Além disso, as plataformas de mídia social têm um papel importante a desempenhar na redução da disseminação de desinformação, implementando normas mais robustas de moderação de conteúdo, promovendo fontes confiáveis de informação ^34^. A seguir, discutiremos mais sobre vetores de desinformação identificados.

### Vetores de desinformação (C2)

Na categoria “vetores de desinformação” foram analisados 30 comentários. Por “vetores de desinformação” entendemos os comentários de usuários que, além de conter algum conteúdo desinformativo que reflete o processo de polarização política da vacina, oferecem links de direcionamentos para outros portais e sites com fontes de informação duvidosas ou inverídicas, ampliando assim o alcance da desinformação. Em outras palavras, esses usuários, a partir de comentários desinformativos, auxiliam ainda mais a propagação de desinformação ao fornecer links externos às reportagens. A análise desses comentários destaca a variedade de formas pelas quais a desinformação pode ser disseminada, incluindo críticas políticas, teorias da conspiração e ataques à mídia ^35^. No Quadro 2, optamos por não incluir os links para os portais desinformativos, assumindo uma postura ético-política em não gerar mais visibilidade e engajamento a tais sites, apenas mencionamos para qual site/portal o link redirecionava entre colchetes. Os sites externos promoviam teorias da conspiração, falsas alegações sobre vacinas ou informações não verificadas sobre a dengue. A presença desses vetores de desinformação destaca a importância de entender não apenas o conteúdo dos comentários, mas também os canais pelos quais a desinformação está sendo disseminada. Ao analisar os links de direcionamento presentes nos comentários, podemos identificar as fontes de desinformação mais prevalentes e compreender melhor como o público está sendo exposto a informações incorretas sobre saúde.

Os comentários analisados expressam uma variedade de opiniões e críticas, mas não fornecem informações concretas sobre a vacinação. Em vez disso, eles direcionam críticas ao governo, à imprensa e a figuras políticas específicas, como o Presidente da República. Esses comentários demonstram como a desinfodemia atua em plataformas de mídia social, em que opiniões políticas e críticas são misturadas com informações sobre temas de grande relevância em saúde pública, sem embasamento factual ^36^ ^,^ ^37^. Compreender esses vetores pode ser essencial na identificação dos elementos desinformativos na percepção pública, auxiliando as autoridades de saúde e os especialistas em comunicação a desenvolver estratégias mais eficazes para combater *fake news* com informações precisas, direcionando esforços para a educação popular em saúde, entre outros ^38^ ^,^ ^39^. Além disso, a identificação de vetores de desinformação também destaca a necessidade de uma abordagem colaborativa e multifacetada para combater a desinformação. Isso inclui a responsabilização das plataformas de mídia social na moderação de conteúdo, a promoção da alfabetização digital e em saúde entre os usuários e a promoção de parcerias entre as autoridades de saúde, organizações da sociedade civil e meios de comunicação para promover fontes assertivas ^40^. Ao compreender como os caminhos pelo qual a desinfodemia age, nos aproximamos do desenvolvimento de estratégias mais eficazes para promover uma sociedade mais bem informada, cidadã e democrática.

### Polarização política da vacina (C3)

A polarização política das vacinas é um fenômeno que permeia o espaço público da saúde no Brasil, gerando debates acalorados e polarizados sobre sua eficácia e segurança, como foi visto no caso da pandemia de COVID-19 ^41^. Esta é a categoria de análise que mais reuniu comentários durante a investigação, um total de 232, revelando que a polarização da sociedade brasileira ainda é um problema atual. Contudo, esta categoria se diferencia da de “vetores de desinformação”, já que aqui os usuários apenas expõem sua opinião, mas não necessariamente induzem outros usuários a links externos desinformativos.

Questões ideológicas e partidárias muitas vezes tornam turva a compreensão da sociedade sobre ciência, levando a uma atmosfera de desconfiança e controvérsia em torno de novos procedimentos e tecnologias. As opiniões sobre vacinas em geral são frequentemente moldadas por afiliações políticas, o que pode distorcer a percepção pública e minar os esforços realizados no âmbito da saúde pública ^42^. A polarização política da vacina contra a dengue também pode resultar em uma seletividade de vacinação, na qual a aceitação ou recusa da vacina é influenciada mais pela afinidade político-partidária do que pela avaliação racional da segurança de uma vacina incorporada ao SUS. Esse é um dos elementos que permitem entender que ainda é presente no Brasil uma sociedade polarizada politicamente após um longo período de instabilidade institucional ^36^. Destaca-se que tal fenômeno relaciona-se intimamente à polarização política das vacinas contra a COVID-19 durante o governo Bolsonaro. Observa-se que muitos dos comentários críticos são feitos por apoiadores de Bolsonaro, que manifestam desconfiança não apenas em relação às vacinas em geral, mas também naquelas promovidas pelo governo subsequente do Partido dos Trabalhadores (PT). Esse cenário reflete um padrão de comportamento similar ao observado durante a crise da COVID-19, com os mesmos grupos expressando ceticismo radical. Essa continuidade na polarização política das vacinas é um analisador fundamental, dado o alto volume de comentários nessa categoria em comparação com outras.

Nessa categoria identificamos um ambiente propício para a propagação de desinformação e teorias da conspiração, além de debates inflamados politicamente. Não existe caminho consolidado para combater o problema, mas é fundamental promover espaços para o diálogo e comunicação baseada em evidências robustas, uma aposta na participação social e cidadã. Nesse sentido, não somente os líderes políticos, mas também as instituições, os profissionais de saúde e os meios de comunicação têm um papel crucial a desempenhar na despolarização do debate sobre vacinas, por exemplo. Ao abordar as preocupações legítimas do público de forma objetiva e acessível, é possível construir um caminho mais profícuo para que exista apoio massivo à vacinação não somente contra a dengue, mas também outras doenças ^37^. Na próxima seção, discutiremos especificamente questões sobre hesitação e recusa vacinal identificadas no estudo.

### Hesitação vacinal e recusa vacinal (C4)

Nessa categoria foram codificados 94 comentários. A hesitação vacinal pode ser caracterizada pelo atraso ou até mesmo a recusa em vacinar-se, uma vez que os imunizantes estão disponíveis no sistema de saúde ^37^. É, sobretudo, um desafio complexo e multifatorial que envolve desde crenças pessoais até a confiança na ciência e nos sistemas de saúde ^29^. A hesitação vacinal afeta a eficácia das campanhas de vacinação contra a dengue e outras doenças ^30^ ^,^ ^31^. A hesitação e/ou recusa vacinal identificada na pesquisa inclui a desconfiança nas autoridades de saúde, preocupações sobre os possíveis efeitos colaterais das vacinas e influência de informações falsas ou enganosas. Para muitas pessoas, a decisão de receber uma vacina é complexa e influenciada por uma série de crenças, experiências passadas e influências sociais.

A literatura destaca que abordar a hesitação vacinal requer uma estratégia que reconheça e respeite as preocupações e dúvidas individuais das pessoas. No entanto, do ponto de vista coletivo, essa abordagem é estratégica para o país ^43^. Isso inclui fornecer informações claras e precisas sobre a vacina da dengue, seus benefícios e riscos, bem como abordar ativamente mitos e desinformação que possam estar circulando. Além disso, destaca-se a importância de envolver a comunidade e os líderes locais no processo de educação e promoção da vacinação, criando um ambiente de confiança e apoio à imunização, fomentando o engajamento e garantindo as metas de cobertura vacinal. Isso não apenas protege indivíduos e comunidades contra doenças evitáveis, mas também contribui para que os sistemas de saúde sejam menos sobrecarregados e mais eficientes ^44^.

### Experiências pessoais (C5)

Ao todo, foram codificados 42 comentários nessa categoria. As experiências pessoais desempenham um papel fundamental na forma como as pessoas percebem e respondem a campanhas de saúde pública, seja por acreditar na gravidade da doença, seja por experiências próprias ou de amigos e familiares que as sensibilizaram para o tema ^45^. Histórias de sucesso e relatos positivos de indivíduos que foram vacinados podem aumentar a confiança na vacina e incentivar outros a buscar a imunização. Por outro lado, experiências negativas ou relatos de eventos adversos à medicação podem gerar receio ou recusa em relação à vacinação ^46^.

É importante reconhecer e respeitar as experiências individuais das pessoas ao abordar questões relacionadas à vacina, pois essas experiências são carregadas com a visão subjetiva de quem recebe a informação. Exemplo disso é um estudo de 2023 que analisou os sentimentos diante das primeiras notícias da chegada do imunizante Qdenga naquele ano. O estudo identificou que, de quase 15 mil *tweets*, cerca de 2 mil eram positivos, 9 mil eram negativos e 4 mil foram considerados neutros ^4^. Nesse sentido, os profissionais da saúde podem ouvir as preocupações, os sentimentos e as dúvidas dos usuários, fornecendo informações factíveis sobre a vacina, auxiliando que a tomada de decisão seja bem informada ^20^. Além disso, compartilhar histórias de sucesso e testemunhos de indivíduos que foram vacinados com segurança pode ajudar a dissipar mitos e desinformação e construir confiança na vacinação, com base no respeito mútuo e solidariedade ^31^. Isso fortalece não apenas os programas de vacinação contra a dengue, mas também a saúde pública, garantindo acesso a informações e serviços de saúde de qualidade, fundamental para a defesa do direito à saúde ^44^ ^,^ ^46^.

## Considerações finais

Ao longo deste estudo, foi possível observar que a polarização política das vacinas, a hesitação vacinal e as experiências pessoais desempenham papéis significativos na forma como as pessoas percebem e respondem à imunização. A polarização do tema, muitas vezes permeada por ideologias políticas e partidárias, pode distorcer a percepção pública e minar os esforços de saúde pública. Além disso, a hesitação vacinal e a recusa vacinal, influenciada por uma série de fatores como desconfiança nas autoridades de saúde e disseminação de desinformação, podem comprometer a eficácia das campanhas de vacinação. No entanto, também reconhecemos que as experiências pessoais desempenham um papel crucial na decisão de se vacinar. Histórias de sucesso e relatos positivos podem aumentar a confiança na vacina, enquanto experiências negativas podem gerar dúvidas e hesitação. 

Para enfrentar esses desafios, é mister um esforço conjunto de diversos setores da sociedade, incluindo governos, profissionais de saúde, mídia e comunidade. Nesse sentido, a promoção de uma comunicação transparente e adaptada aos contextos locais é fundamental para construir confiança e aumentar a adesão à vacinação contra a dengue. Por fim, ressalta-se que uma limitação deste estudo está na rede social analisada, porque reflete apenas uma população específica que faz o uso da rede social Facebook, sendo importante compreender também os elementos desinformativos na percepção pública sob outros prismas. Destaca-se também a grande produção de literatura na temática sobre desinformação e vacinas, uma resposta importante dada pela ciência diante do negacionismo vivenciado na pandemia de COVID-19 e que ainda reverbera no espaço público, o que é relevante. Refletir e aprender com experiências passadas é essencial para avançar nessa discussão. Por fim, a identificação de elementos de desinformação na percepção pública revela a importância da comunicação em saúde para a implementação de políticas, sua aceitação e adesão, contribuindo assim para a redução do impacto da doença na saúde pública.
